# Detailed behaviour of endothelial wall shear stress across coronary lesions from non-invasive imaging with coronary computed tomography angiography

**DOI:** 10.1093/ehjci/jeac095

**Published:** 2022-05-25

**Authors:** Inge J van den Hoogen, Jussi Schultz, Jurrien H Kuneman, Michiel A de Graaf, Vasileios Kamperidis, Alexander Broersen, J Wouter Jukema, Antonis Sakellarios, Sotirios Nikopoulos, Savvas Kyriakidis, Katerina K Naka, Lampros Michalis, Dimitrios I Fotiadis, Teemu Maaniitty, Antti Saraste, Jeroen J Bax, Juhani Knuuti

**Affiliations:** Department of Cardiology, Leiden University Medical Center, Leiden, The Netherlands; Turku PET Centre, Turku University Hospital and University of Turku, Kiinamyllynkatu 4-8, Turku 20520, Finland; Department of Cardiology, Leiden University Medical Center, Leiden, The Netherlands; Department of Cardiology, Leiden University Medical Center, Leiden, The Netherlands; Department of Cardiology, Leiden University Medical Center, Leiden, The Netherlands; Department of Radiology, Division of Image Processing, Leiden University Medical Center, Leiden, The Netherlands; Department of Cardiology, Leiden University Medical Center, Leiden, The Netherlands; Department of Biomedical Research, FORTH-IMBB, Ioannina, Greece; Unit of Medical Technology and Intelligent Information Systems, Department of Materials Science and Engineering, University of Ioannina, Ioannina, Greece; Department of Cardiology, Medical School, University of Ioannina, Ioannina, Greece; Department of Biomedical Research, FORTH-IMBB, Ioannina, Greece; Unit of Medical Technology and Intelligent Information Systems, Department of Materials Science and Engineering, University of Ioannina, Ioannina, Greece; Department of Cardiology, Medical School, University of Ioannina, Ioannina, Greece; Department of Cardiology, Medical School, University of Ioannina, Ioannina, Greece; Department of Biomedical Research, FORTH-IMBB, Ioannina, Greece; Unit of Medical Technology and Intelligent Information Systems, Department of Materials Science and Engineering, University of Ioannina, Ioannina, Greece; Turku PET Centre, Turku University Hospital and University of Turku, Kiinamyllynkatu 4-8, Turku 20520, Finland; Turku PET Centre, Turku University Hospital and University of Turku, Kiinamyllynkatu 4-8, Turku 20520, Finland; Heart Center, Turku University Hospital and University of Turku, Turku, Finland; Department of Cardiology, Leiden University Medical Center, Leiden, The Netherlands; Heart Center, Turku University Hospital and University of Turku, Turku, Finland; Department of Cardiology, Leiden University Medical Center, Leiden, The Netherlands; Turku PET Centre, Turku University Hospital and University of Turku, Kiinamyllynkatu 4-8, Turku 20520, Finland

**Keywords:** atherosclerosis, coronary artery disease, endothelial wall shear stress, computational fluid dynamics, coronary computed tomography angiography

## Abstract

**Aims:**

Evolving evidence suggests that endothelial wall shear stress (ESS) plays a crucial role in the rupture and progression of coronary plaques by triggering biological signalling pathways. We aimed to investigate the patterns of ESS across coronary lesions from non-invasive imaging with coronary computed tomography angiography (CCTA), and to define plaque-associated ESS values in patients with coronary artery disease (CAD).

**Methods and results:**

Symptomatic patients with CAD who underwent a clinically indicated CCTA scan were identified. Separate core laboratories performed blinded analysis of CCTA for anatomical and ESS features of coronary atherosclerosis. ESS was assessed using dedicated software, providing minimal and maximal ESS values for each 3 mm segment. Each coronary lesion was divided into upstream, start, minimal luminal area (MLA), end and downstream segments. Also, ESS ratios were calculated using the upstream segment as a reference. From 122 patients (mean age 64 ± 7 years, 57% men), a total of 237 lesions were analyzed. Minimal and maximal ESS values varied across the lesions with the highest values at the MLA segment [minimal ESS 3.97 Pa (IQR 1.93–8.92 Pa) and maximal ESS 5.64 Pa (IQR 3.13–11.21 Pa), respectively]. Furthermore, minimal and maximal ESS values were positively associated with stenosis severity (*P* < 0.001), percent atheroma volume (*P* < 0.001), and lesion length (*P* ≤ 0.023) at the MLA segment. Using ESS ratios, similar associations were observed for stenosis severity and lesion length.

**Conclusions:**

Detailed behaviour of ESS across coronary lesions can be derived from routine non-invasive CCTA imaging. This may further improve risk stratification.

## Graphical Abstract

**Graphical Abstract:**
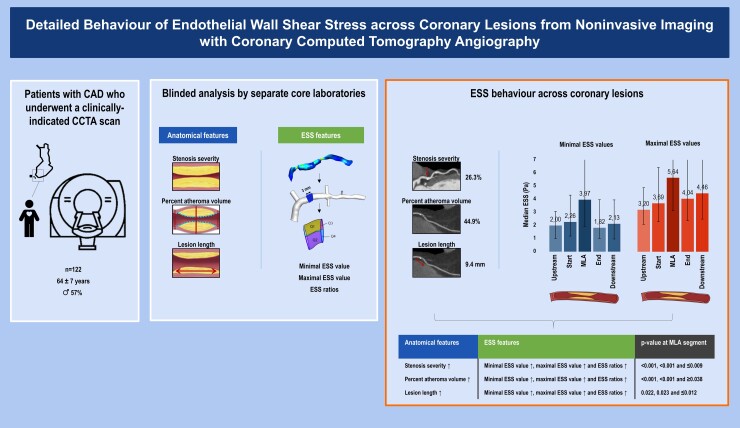
Schematic representation of the study design and patients (left panel), the comprehensive image analysis process including blinded analysis of anatomical and ESS features of coronary atherosclerosis (middle panel), and the patterns of ESS across lesions (right panel). Images are partly derived and adjusted from Ohayon *et al.*, Biomechanics of coronary atherosclerotic plaque: from model to patient, Academic Press, 1st edition, chapter 9, page 207, copyright 2020 and Puchner *et al.*, High-risk plaque detected on coronary CT angiography predicts acute coronary syndromes independent of significant stenosis in acute chest pain: results from the ROMICAT-II trial, Journal of the American College of Cardiology, volume 64, page 687, copyright 2014, both with permission from Elsevier.^31,32^ CAD, coronary artery disease; CCTA, coronary computed tomography angiography; ESS, endothelial wall shear stress.

## Introduction

Endothelial wall shear stress (ESS) is a biomechanical stress that develops from frictional forces of blood flow against the vessel wall in vascular tissue, such as the coronary arteries.^1^ ESS is relatively small in orders of magnitude, but can uniquely trigger biological signalling pathways involved in the natural history of coronary artery disease (CAD).^2,3^ It has been postulated that ESS plays a pivotal role in the rupture and progression of individual coronary lesions through stimulating fibrous cap thinning and inducing local inflammation, respectively.^4,5^ Hence, ESS is a hypothesized marker of rupture-vulnerable plaque that has the potential to refine and enhance the risk stratification of lesions. Although prior research has evaluated this specific role of ESS, most studies have been limited to invasive imaging techniques in high-risk patients with CAD.^6–8^ Coronary computed tomography angiography (CCTA) has rapidly emerged as the routine tool to non-invasively evaluate and characterize coronary atherosclerosis with excellent diagnostic certainty.^9,10^ Recently, technological advancements in CCTA imaging have enabled the non-invasive assessment of ESS.^11–13^ Therefore, the primary aim of this study was to investigate the detailed behaviour of ESS across coronary lesions from non-invasive CCTA imaging in patients with CAD. Secondary aims included the definition of ESS values associated with anatomical features of coronary atherosclerosis.

## Methods

### Study design and patients

Consecutive symptomatic patients with suspected CAD and a clinical indication for CCTA were prospectively enrolled at the Turku University Hospital, Turku, Finland between 2007 and 2011. All patients had an intermediate pre-test likelihood of obstructive CAD. The study design has been published earlier in detail.^14^ Of those enrolled, a total of 549 vessels from 183 patients underwent blinded analysis of anatomical and ESS features of coronary atherosclerosis by separate core laboratories. The study protocol was approved by the ethics committee of the Hospital District of South-West Finland, and the need for written informed consent was waived. The study was conducted in direct compliance with the Declaration of Helsinki. For the current analysis, vessels with failed analysis of anatomical features (*n* = 37), failed analysis of ESS features (*n* = 93), impaired image quality (*n* = 22), chronic total occlusions (*n* = 30), unfeasible co-registration (*n* = 38), or absence of disease (*n* = 141) were excluded. Hence, for our analysis 188 vessels and 237 lesions from 122 patients with CAD were included.

### CCTA acquisition and image analysis

Patients were scanned with a 64-detector row positron emission tomography/CT scanner (GE Discovery VCT or GE D690, General Electric Medical Systems, Waukesha, WI, USA), and detailed protocols regarding the acquisition of scans were previously reported.^14,15^ Protocols included the systematic administration of intravenous metoprolol (0–30 mg), sublingual nitroglycerin (800 μg), or isosorbide dinitrate (1.25 mg) before acquisition to achieve target heart rates (<60/min) and coronary vasodilatation, respectively.^14^ If feasible, prospectively triggered acquisition was employed in an attempt to reduce radiation dose.

#### Analysis of anatomical features

Scans were quantitatively analyzed by 1 independent reader (V.K.) for anatomical features at the Dutch core laboratory (Leiden University Medical Center, Leiden, the Netherlands) according to the 17-segment modified American Heart Association model, blinded to clinical and ESS results.^16,17^ All coronary segments ≥1.5 mm in diameter were evaluated. Quantitative analysis was assessed using semi-automated validated software (QAngio CT Research Edition version 1.3.6, Medis Medical Imaging Systems, Leiden, the Netherlands) with manual correction if needed.^18^ Coronary lesions were defined as tissue ≥1 mm^2^ within or adjacent to the coronary lumen discriminable in >2 planes from pericardial tissue, epicardial fat, or lumen.^19^ For each coronary lesion, measurement of maximal diameter stenosis, percent atheroma volume, and lesion length were completed. Maximal diameter stenosis was graded by severity into <25%, 25–50%, and ≥50%. Percent atheroma volume was calculated according to standardized definitions, and categorized into small (<44.9%) and large (≥44.9%) using the mean as a cut-off.^20^ Lesion length was categorized into short (<9.4 mm) and long (≥9.4 mm), using the median as a threshold. All measurements were performed on a per-segment and per-lesion basis and summation of these values produced per-patient data.

#### Analysis of ESS features

Scans were separately analyzed by 2 independent readers (S.N. and L.M.) for ESS features at the Greek core laboratory (University of Ioannina, Ioannina, Greece), blinded to clinical and anatomical results. All coronary segments of the main epicardial arteries [i.e. left anterior descending artery (LAD), left circumflex artery (LCx), right coronary artery (RCA)] were evaluated, except for their corresponding side branches.^21^ Notably, the left main artery (LM) was analyzed as part of the LAD. ESS was assessed using dedicated software (SMARTool version 0.9.17, FORTH, Ioannina, Greece) by the following consecutive steps.^22–24^ First, three-dimensional reconstructions were created of each vessel in the shape of a tetrahedral mesh.^22,25^ Second, steady-state flow simulations were performed using finite element software (ANSYS CFX version 18.1, Canonsburg, Philadelphia, PA, USA) for the solution of Navier-Stokes and continuity equations.^21^ To this end, specific boundary conditions were applied. The vessel wall was assumed to be rigid with a no-slip condition.^26,27^ For the inlet boundary condition, a fixed mean pressure of 100 mmHg was selected to simulate myocardial blood flow at a resting state. For the outlet boundary condition, a typical outflow coronary uniform velocity profile of 1 mL/s was selected to describe a resting state.^28,29^ Blood was assumed to be a Newtonian fluid with a dynamic viscosity of 0.0035 Pa×s and a density of 1050 kg/m^3^. The flow was assumed to be laminar and incompressible. Third, ESS was calculated at the luminal surface of each vessel as the product of viscosity and the gradient of blood velocity near the vessel wall.^21^ Fourth, ESS was extracted by employing the segmental method, providing minimal and maximal ESS values for each 3 mm segment (see Supplementary data online, *Figure S1*).^30,31^ The segmental method divided each 3 mm segment into quarter cylinders with a 90° arc, and for the separate cylinders the ESS over the luminal surface was averaged. To assess the minimal ESS value, the minimum averaged value of the cylinders was assigned to the entire segment. Likewise, the maximal averaged value was assigned to the entire segment to determine the maximal ESS value. For each vessel, measurement of minimal and maximal ESS values was completed over its total length.

#### Co-registration and consensus reads

Visual co-registration of vessel centrelines from the anatomical and ESS output was performed using fiduciary landmarks to determine the exact location position (i.e. 3 mm segment) of coronary lesions by consensus of ≥3 experienced readers (A.S., I.J.v.d.H., J.H.K., J.K., J.S.) (see Supplementary data online, *Figure S2*). The interval between the consensus reads was 2–3 months. For each coronary lesion, 5 locations of interest were identified: (i) upstream segment, (ii) start segment, (iii) minimal luminal area (MLA) segment, (iv) end segment, and (v) downstream segment. Note that locations 2–4 could be located in a single 3 mm segment in case of small and/or short lesions. Intraobserver intraclass correlation coefficient for locations was 0.934 (*P* < 0.001) in 47 (20%) randomly selected lesions. For all locations, minimal and maximal ESS ratios were calculated as: minimal or maximal ESS value at the segment of that specific location/minimal or maximal ESS value at the upstream segment. Therefore, these ratios were always 1.0 at the upstream segment and represented the relative change in ESS as compared with this reference.

### Statistical analysis

Continuous data are presented as means ± standard deviations or medians with interquartile ranges (IQR) on the basis of their distribution. Categorical data are presented as counts with percentages. Continuous data were compared with the Independent-Samples *T* test or Mann–Whitney *U* test (for 2 group comparisons), and the Kruskal–Wallis test (for 3 group comparisons). Categorical data were compared with the *χ*^2^ test. For visual interpretation, bar charts were created. Coronary lesions were treated as independent observations for the purpose of this analysis. All statistical tests were two-sided and a *P*-value of <0.05 specified statistical significance. All analyses were performed with SPSS software (version 26, SPSS IBM Corp., Armonk, NY, USA).

## Results

### Patient characteristics

Baseline patient characteristics are depicted in *Table 1*. A total of 122 patients with CAD (mean age 64 ± 7 years, 57% men) underwent blinded analysis of CCTA for anatomical and ESS features of coronary atherosclerosis. All patients were symptomatic with prevalent cardiac risk factors and medication use. In particular, a high prevalence of hypertension (74%) and dyslipidaemia (74%) was observed.

**Table 1:** Baseline patient characteristics

	Patients with ≥1 coronary lesion *n* = 122 Mean ± SD or *n* (%)
Age, years	64 ± 7
Male	70 (57)
BMI, kg/m^2^	28.1 ± 4.7
*Symptoms*	
Typical angina	38 (33)
Atypical angina	57 (49)
Non-cardiac pain	21 (18)
Dyspnoea at exertion	42 (50)
*Cardiac risk factors*	
Hypertension	90 (74)
Dyslipidaemia	90 (74)
Diabetes mellitus	26 (21)
Family history of CAD	54 (44)
Smoking current or former	48 (44)
*Cardiac medication*	
Aspirin	79 (74)
Beta blockers	67 (63)
Calcium channel blockers	20 (19)
Renin–angiotensin system inhibitors	33 (31)
Statins	64 (59)
*Laboratory findings*	
Total cholesterol, mmol/L	4.8 ± 1.0
Low-density lipoprotein, mmol/L	2.6 ± 0.8
High-density lipoprotein, mmol/L	1.6 ± 0.4
Triglycerides, mmol/L	1.4 ± 0.8
Creatinine, µmol/L	76.8 ± 16.8

BMI, body mass index; CAD, coronary artery disease.

### Anatomical characteristics of coronary lesions

Anatomical characteristics of the lesions are depicted in *Table 2*. For the analyzed 237 lesions, mean maximal diameter stenosis was 26.3 ± 15.2%, mean percent atheroma volume was 44.9 ± 10.3%, and median lesion length was 9.4 mm (IQR 5.0–15.5 mm). Most lesions were located in the LM or LAD (58%), followed by the RCA (22%) and LCx (21%). More specifically, the location within the vessel was proximal for the vast majority (77%), followed by mid (19%) and distal (3%).

**Table 2:** Anatomical characteristics of lesions

	Coronary lesions *n* = 237 Mean ± SD, median (IQR) or *n* (%)
*General*	
Maximal diameter stenosis, %	26.3 ± 15.2
Maximal cross-sectional plaque burden, %	60.0 ± 15.9
Percent atheroma volume, %	44.9 ± 10.3
Plaque volume, mm^3^	57.5 (31.3–110.0)
Lesion length, mm	9.4 (5.0–15.5)
*Vessel location*	
LM, LAD	137 (58)
RCA	51 (22)
LCx	49 (21)
*Within-vessel location*	
Proximal	183 (77)
Mid	46 (19)
Distal	8 (3)

LAD, left anterior descending artery; LCx, left circumflex artery; LM, left main artery; RCA, right coronary artery.

### ESS characteristics of coronary lesions

#### Absolute ESS values

**Figure 1:**
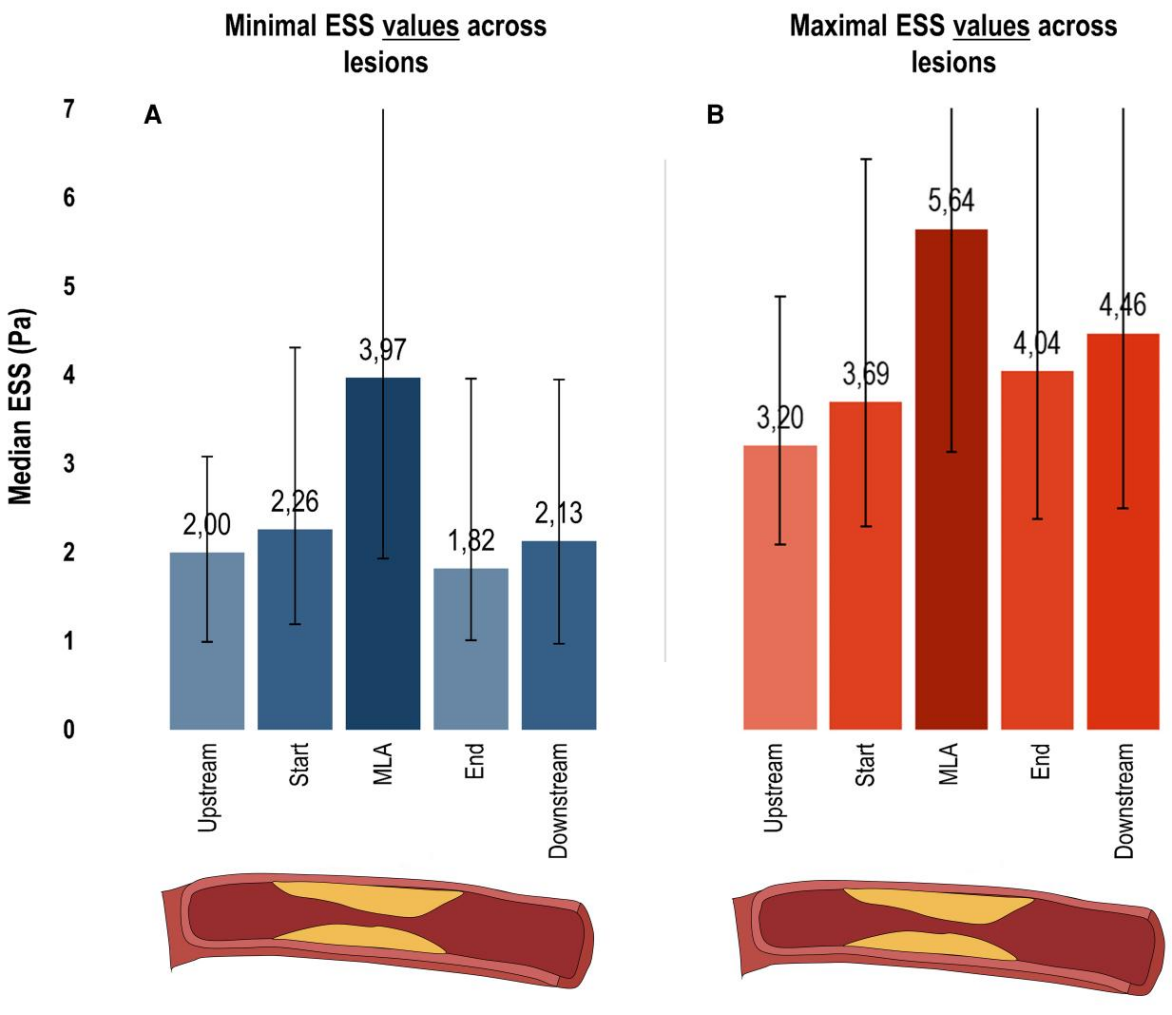
ESS characteristics of coronary lesions. Bar charts depict the median minimal (*A*) and maximal (*B*) ESS values with IQR bars (*y*-axis: increased values = increased median ESS in Pa) for 5 locations across the 237 lesions (*x*-axis; upstream segment, start segment, MLA segment, end segment, downstream segment). ESS, endothelial wall shear stress; MLA, minimal luminal area.

Minimal ESS values as related to each lesion location are visually presented in *Figure 1A*. Median minimal ESS value was 2.00 Pa (IQR 0.99–3.08 Pa) at the upstream segment, and thereafter increased to 2.26 Pa (IQR 1.19–4.31 Pa) at the start segment and 3.97 Pa (IQR 1.93–8.92 Pa) at the MLA segment. After the MLA, values decreased to 1.82 Pa (IQR 1.01–3.96 Pa) at the end segment and increased again to 2.13 Pa (IQR 0.97–3.95 Pa) at the downstream segment. Similarly, the median maximal ESS value was 3.20 Pa (IQR 2.09–4.88 Pa) at the upstream segment, and raised to 3.69 Pa (IQR 2.29–6.43 Pa) at the start segment and 5.64 Pa (IQR 3.13–11.21 Pa) at the MLA segment (*Figure 1B*). After this point, values reduced to 4.04 Pa (IQR 2.38–7.57 Pa) at the end segment and marginally raised to 4.46 Pa (IQR 2.49–7.57 Pa) at the downstream segment.

#### Relative ESS values

**Figure 2:**
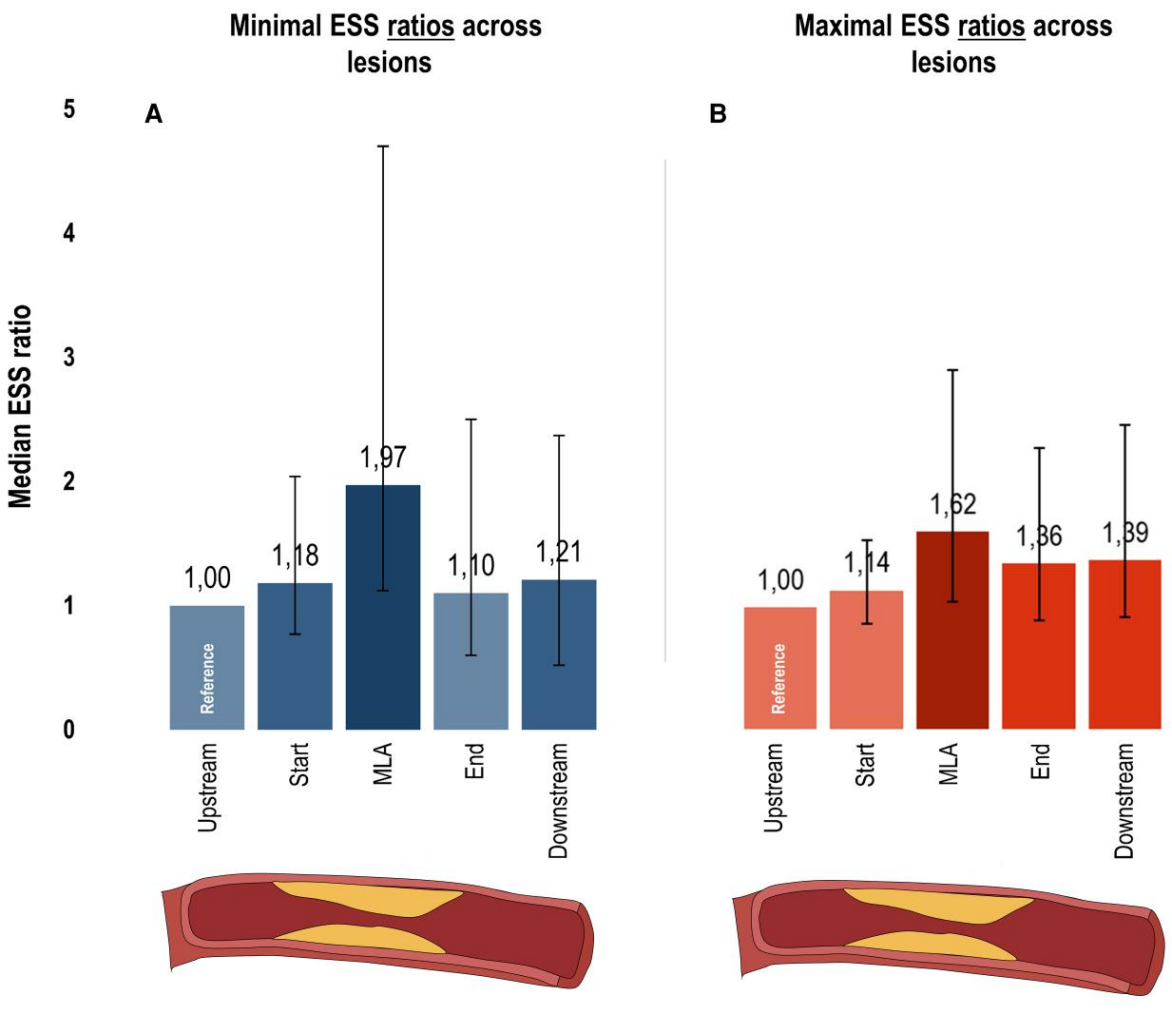
ESS characteristics of coronary lesions using ratios. Bar charts depict the median minimal (*A*) and maximal (*B*) ESS ratios with IQR bars (*y*-axis: increased values = increased median ratio) for 5 locations across the 237 lesions (*x*-axis; upstream segment, start segment, MLA segment, end segment, downstream segment). Minimal and maximal ESS ratios were calculated as: minimal or maximal ESS value at the segment of that specific location/minimal or maximal ESS value at the upstream segment. Hence, these ratios were always 1.0 at the upstream segment (i.e. reference). ESS, endothelial wall shear stress; MLA, minimal luminal area.

Minimal ESS ratios as related to each lesion location are displayed in *Figure 2A*. Median minimal ESS ratio was 1.18 (IQR 0.77–2.04) at the start segment and peaked to 1.97 (IQR 1.12–4.70) at the MLA segment. After the MLA, ratios decreased to 1.10 (IQR 0.60–2.50) at the end segment and slightly increased to 1.21 (IQR 0.52–2.37) at the downstream segment. Likewise, the median maximal ESS ratio was 1.14 (IQR 0.87–1.55) at the start segment and raised to 1.62 (IQR 1.05–2.95) at the MLA segment (*Figure 2B*). After this point, ratios reduced to 1.36 (IQR 0.89–2.31) at the end segment and minimally raised to 1.39 (IQR 0.92–2.50) at the downstream segment.

### Association between ESS and anatomical features

On a per-lesion level, minimal and maximal ESS values were positively associated with stenosis severity, mainly at the MLA segment (both *P* < 0.001) (*Table 3*). At the MLA, median minimal ESS values varied from 2.80 Pa (IQR 1.66–5.70 Pa) for <25% stenosis to 11.00 Pa (IQR 3.26–28.55 Pa) for ≥50% stenosis. Likewise, median maximal ESS values varied from 4.21 Pa (IQR 2.45–8.41 Pa) for <25% stenosis to 14.55 Pa (IQR 4.32–32.34 Pa) for ≥50% stenosis. Positive associations were also observed for large versus small percent atheroma volume (*P* < 0.001) and long versus short lesion length (*P* ≤ 0.023) at least at the MLA segment (*Tables 4* and *5*). Comparably, minimal and maximal ESS ratios were positively associated with stenosis severity (*P* = 0.009 and *P* = 0.004) and lesion length (*P* = 0.007 and *P* = 0.012) at the MLA segment (see Supplementary data online, *Tables S1–3*). However, only maximal ESS ratios were positively associated with percent atheroma volume (*P* = 0.038) at this location.

**Table 3:** Per-lesion ESS values according to stenosis severity

	<25% stenosis *n* = 129 Median (IQR)	25–50% stenosis *n* = 89	≥50% stenosis *n* = 19	*P*-value	*P*-value for trend
*Minimal values*					
ESS_upstream_, Pa	2.01 (0.91–2.99)	1.86 (1.10–3.12)	3.24 (0.93–5.54)	0.293	0.262
ESS_start_, Pa	2.24 (1.00–4.14)	2.38 (1.38–4.40)	2.33 (0.72–5.05)	0.523	0.328
ESS_MLA_, Pa	2.80 (1.66–5.70)	4.94 (2.61–10.61)	11.00 (3.26–28.55)	<0.001	<0.001
ESS_end_, Pa	1.67 (1.05–3.01)	1.83 (0.97–5.30)	3.71 (0.94–13.40)	0.094	0.055
ESS_downstream_, Pa	2.04 (0.95–3.65)	2.26 (1.04–4.31)	2.78 (1.05–7.12)	0.249	0.101
*Maximal values*					
ESS_upstream_, Pa	3.08 (1.85–4.56)	3.27 (2.29–4.76)	6.45 (1.98–9.14)	0.068	0.030
ESS_start_, Pa	3.62 (1.75–5.81)	3.70 (2.51–6.01)	6.68 (2.40–13.89)	0.162	0.079
ESS_MLA_, Pa	4.21 (2.45–8.41)	6.59 (3.73–14.32)	14.55 (4.32–32.34)	<0.001	<0.001
ESS_end_, Pa	3.41 (2.13–6.23)	4.65 (2.54–9.14)	9.69 (4.58–33.76)	<0.001	<0.001
ESS_downstream_, Pa	3.85 (2.13–7.17)	4.64 (2.54–7.89)	8.47 (4.14–16.58)	0.014	0.009

ESS, endothelial wall shear stress; MLA, minimal luminal area.

**Table 4:** Per-lesion ESS values according to volumetric plaque burden

	Small percent atheroma volume *n* = 116 Median (IQR)	Large percent atheroma volume *n* = 121	*P*-value
*Minimal values*			
ESS_upstream_, Pa	1.88 (0.91–2.93)	2.16 (1.07–3.32)	0.160
ESS_start_, Pa	1.98 (1.00–3.68)	2.54 (1.43–4.92)	0.028
ESS_MLA_, Pa	2.94 (1.66–6.16)	4.95 (2.47–12.21)	<0.001
ESS_end_, Pa	1.60 (0.97–2.79)	2.30 (1.08–5.73)	0.005
ESS_downstream_, Pa	1.80 (0.88–2.99)	2.65 (1.08–4.96)	0.001
*Maximal values*			
ESS_upstream_, Pa	3.05 (1.77–4.54)	3.64 (2.32–6.11)	0.018
ESS_start_, Pa	3.09 (1.80–4.91)	4.22 (2.62–7.11)	0.004
ESS_MLA_, Pa	4.17 (2.45–8.78)	6.94 (3.69–14.96)	<0.001
ESS_end_, Pa	3.38 (2.13–6.05)	5.40 (2.76–11.03)	0.001
ESS_downstream_, Pa	3.79 (2.44–6.47)	4.94 (2.60–9.75)	0.018

ESS, endothelial wall shear stress; MLA, minimal luminal area.

**Table 5:** Per-lesion ESS values according to lesion length

	Short lesion length *n* = 119 Median (IQR)	Long lesion length *n* = 118	*P*-value
*Minimal values*			
ESS_upstream_, Pa	2.10 (1.13–3.14)	1.86 (0.79–2.97)	0.239
ESS_start_, Pa	2.49 (1.36–5.77)	2.09 (0.98–3.24)	0.007
ESS_MLA_, Pa	3.02 (1.71–8.04)	4.51 (2.26–11.12)	0.022
ESS_end_, Pa	1.71 (1.13–3.52)	1.88 (0.91–5.26)	0.715
ESS_downstream_, Pa	2.14 (1.01–3.64)	2.12 (0.99–5.32)	0.557
*Maximal values*			
ESS_upstream_, Pa	3.19 (2.14–5.28)	3.24 (2.07–4.66)	0.652
ESS_start_, Pa	3.85 (2.34–7.70)	3.54 (1.95–5.16)	0.048
ESS_MLA_, Pa	4.76 (2.74–10.38)	6.17 (3.67–14.88)	0.023
ESS_end_, Pa	3.64 (2.20–6.39)	4.96 (2.59–9.62)	0.028
ESS_downstream_, Pa	4.22 (2.24–6.59)	4.67 (2.53–10.67)	0.063

ESS, endothelial wall shear stress; MLA, minimal luminal area.

## Discussion

The present study evaluated the feasibility of evaluating ESS from non-invasive CCTA imaging in a large cohort of 122 symptomatic patients with CAD. Our findings revealed the detailed behaviour of absolute and relative ESS values across coronary lesions (*Graphical Abstract*).^31,32^ At the MLA segment, these values—minimal ESS value, maximal ESS value, and ESS ratios—were highest and demonstrated a positive association with anatomical features of coronary atherosclerosis, such as stenosis severity and lesion length.

### ESS behaviour over coronary lesions

A couple of studies have reported on the varying behaviour of ESS across lesions within the coronary artery tree.^6,8,12^ In the Prediction of Progression of Coronary Artery Disease and Clinical Outcome using Vascular Profiling of Shear Stress and Wall Morphology (PREDICTION) study, 506 acute coronary syndrome patients that underwent three-vessel intravascular ultrasound (IVUS) at index percutaneous coronary intervention were examined for ESS patterns.^6^ Highest and lowest ESS were most frequently observed at the throat (73%) and 6 mm distal to the throat (60%) of lesions, respectively. Likewise, Stone *et al*.^8^ evaluated 145 non-culprit lesions from 97 acute coronary syndrome patients that underwent IVUS at index percutaneous coronary intervention. Again, the highest ESS most commonly occurred at the MLA segment (59%), whether or not the non-culprit lesion caused an adverse event after median follow-up of 3.4 years (*P* = 0.78). Moreover, the lowest ESS most often occurred 3–9 mm proximal (31%) or distal (24%) to the MLA segment, irrespective of future event status (*P* = 0.87). Using non-invasive imaging, Park *et al.*^12^ analyzed 80 patients with suspected or known CAD that underwent CCTA among other examinations. By including 79 lesions, ESS was highest at the MLA segment as compared with upstream or downstream segments (*P* < 0.001). Our findings, which provided both (i) absolute ESS values (i.e. minimal, maximal) and (ii) relative ESS values (i.e. ratios) were overall consistent with the abovementioned results and further extended this knowledge with the addition of the comprehensive anatomical characterization of coronary atherosclerosis in a large contemporary cohort of patients. Besides, it should be mentioned that CCTA, in comparison to invasive imaging techniques such as IVUS, is able to image the complete coronary artery tree and is not limited by severe lesions that cannot be passed by a catheter.

### ESS and anatomical features of coronary atherosclerosis

To date, only limited studies have reported on the associations between ESS and anatomical features of coronary atherosclerosis.^11,21,33^ Eshtehardi *et al*.^33^ selected 27 stable patients with angina or an abnormal non-invasive stress test that were referred for virtual histology IVUS. By analyzing 3581 IVUS frames, segments with high ESS were shown to be associated with larger cross-sectional plaque burden. More specifically, ESS remained relatively constant in the smaller quartiles of plaque burden, but significantly increased in the largest quartile of cross-sectional plaque burden. Our findings, which utilized percent atheroma volume as a volumetric measure of plaque burden, revealed a similar positive association. Furthermore, from the Determination of Fractional Flow Reserve by Anatomic Computed Tomographic Angiography (DEFACTO) study, Han *et al.*^11^ studied 100 stable patients with suspected or known CAD that were referred for CCTA and subsequent invasive coronary angiography. By including 163 lesions, ESS was positively associated with stenosis severity on CCTA (*P* = 0.002), and numerically with percent atheroma volume (*P* > 0.05) and lesion length (*P* > 0.05). In addition to this, positive associations were observed between ESS and stenosis severity on invasive coronary angiography (*r* = 0.315, *P* < 0.001): 43% stenosis for low ESS, 48% stenosis for intermediate ESS, and 55% stenosis for high ESS. Recently, Kalykakis *et al.*^21^ investigated 53 patients with suspected CAD that underwent CCTA. By analyzing 92 vessels, ESS was positively associated with stenosis severity: mean ESS values varied from 3.3 ± 3.0 Pa for <50% stenosis to 15.1 ± 30.0 Pa for ≥50% stenosis. Similarly, median ESS values varied from 2.80–4.21 Pa for <25% stenosis to 11.00–14.55 Pa for ≥50% stenosis in the present study.

### Clinical implications

At present, the clinical importance of assessing ESS from CCTA imaging has yet to be elucidated. No evidence exists on normal or physiological ESS values from CCTA, and therefore comparisons to such references could not be performed. Also, uniform thresholds regarding pathological ESS values (i.e. low, intermediate, high) are not available in CCTA literature.^11,13^ For example, the majority of thresholds is based on the distribution of ESS values within diverse study cohorts. As a consequence, it remains unclear whether or not those cut-offs may vary according to differences in baseline risk and computational methods. Moreover, as compared with invasive imaging techniques, ESS values derived from CCTA appear to be higher.^6,8^ Although our first findings seem promising, future studies are warranted to determine the true clinical value of ESS. Interestingly, ESS, as a hypothesized marker of vulnerable plaque, could potentially refine and further enhance risk assessment in patients with CAD undergoing non-invasive CCTA imaging.

### Limitations

The current study is not without limitations. First, our study had an observational design with intrinsic limitations such as confounding and selection bias. Moreover, vessels were excluded because the retrospective assessment of ESS failed, often caused by motion or blooming artefacts. Also, vessels with chronic total occlusions were excluded because their (micro)vasculature and flow were considered highly complex. However, exclusion rates are not dissimilar to prior studies using CCTA.^13,21^ Second, the assessment of ESS did not allow for incorporation of side branches, and therefore these effects were not taken into account. Though, we applied a standardized approach that has been employed in several prior reports.^8,34^ Third, plaque composition of coronary atherosclerosis was not (yet) available for this analysis. Last, 3 mm axial segments were used to co-register anatomical and ESS output and to determine the exact location position of coronary lesions. Despite our meticulous methodology including consensus reads by experienced readers, we cannot rule out that errors may have occurred during this time-consuming process.

## Conclusion

It is feasible to evaluate the precise patterns of ESS across coronary lesions from routine non-invasive CCTA imaging. In particular, the highest absolute and relative ESS values and associations with anatomical features of coronary atherosclerosis were observed at the MLA segment. ESS assessment from CCTA may further improve risk stratification.

## Supplementary data

Supplementary data are available at *European Heart Journal* – *Cardiovascular Imaging* online.

## Supplementary Material

jeac095_Supplementary_Data

## Data Availability

Data may be available upon reasonable request to the corresponding author.
