# Supplementary material for: Detailed behaviour of endothelial wall shear stress across coronary lesions from non-invasive imaging with coronary computed tomography angiography
Source: Eur Heart J Cardiovasc Imaging. 2022 May 25;23(12):1708–16. doi: 10.1093/ehjci/jeac095 (PMC10017098; doi:10.1093/ehjci/jeac095)
Supplement: jeac095_Supplementary_Data [file jeac095_supplementary_data.pptx]

## Slide 1
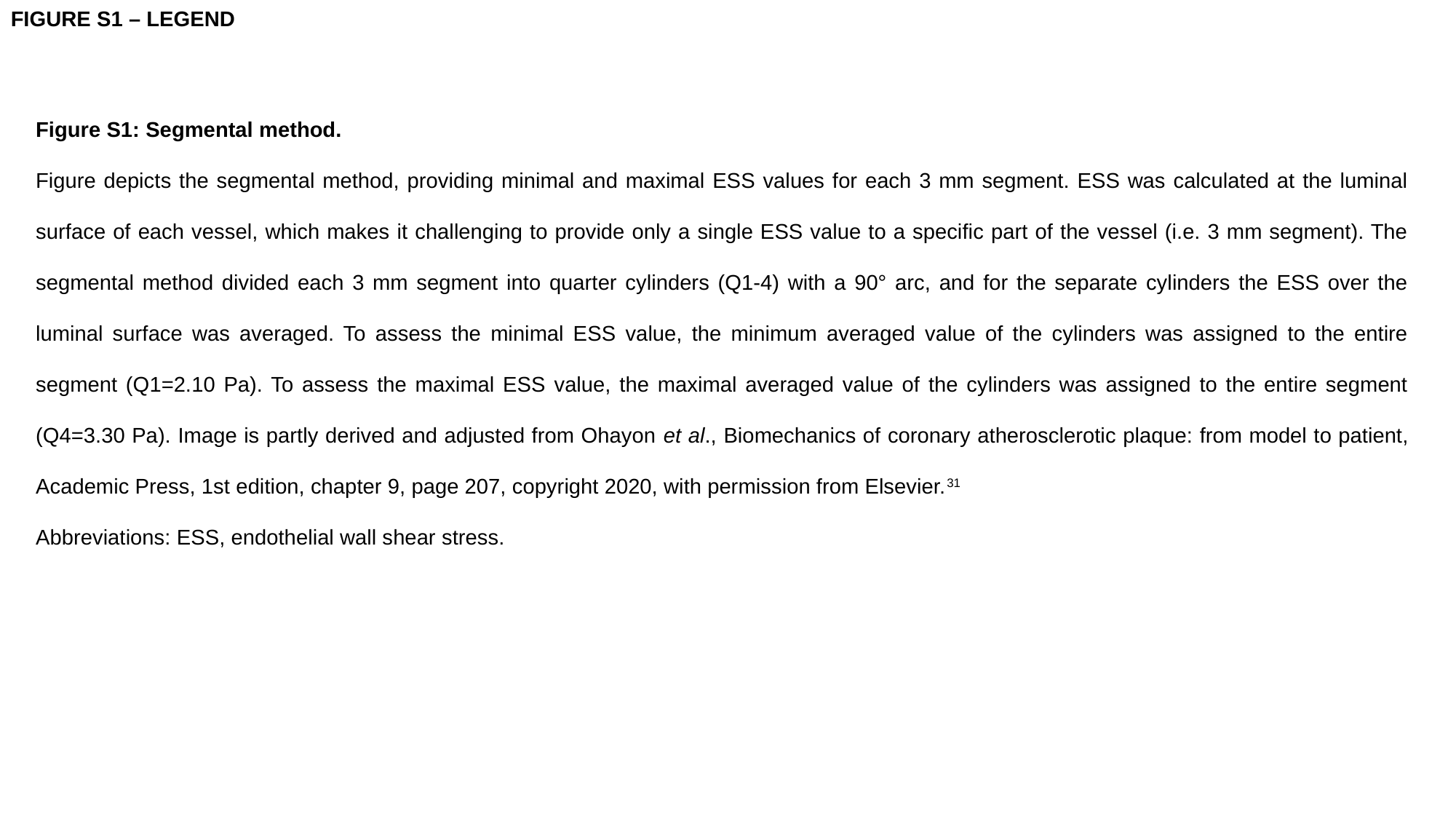

FIGURE S1 – LEGEND
Figure S1: Segmental method.
Figure depicts the segmental method, providing minimal and maximal ESS values for each 3 mm segment. ESS was calculated at the luminal surface of each vessel, which makes it challenging to provide only a single ESS value to a specific part of the vessel (i.e. 3 mm segment). The segmental method divided each 3 mm segment into quarter cylinders (Q1-4) with a 90° arc, and for the separate cylinders the ESS over the luminal surface was averaged. To assess the minimal ESS value, the minimum averaged value of the cylinders was assigned to the entire segment (Q1=2.10 Pa). To assess the maximal ESS value, the maximal averaged value of the cylinders was assigned to the entire segment (Q4=3.30 Pa). Image is partly derived and adjusted from Ohayon et al., Biomechanics of coronary atherosclerotic plaque: from model to patient, Academic Press, 1st edition, chapter 9, page 207, copyright 2020, with permission from Elsevier.31
Abbreviations: ESS, endothelial wall shear stress.

## Slide 2
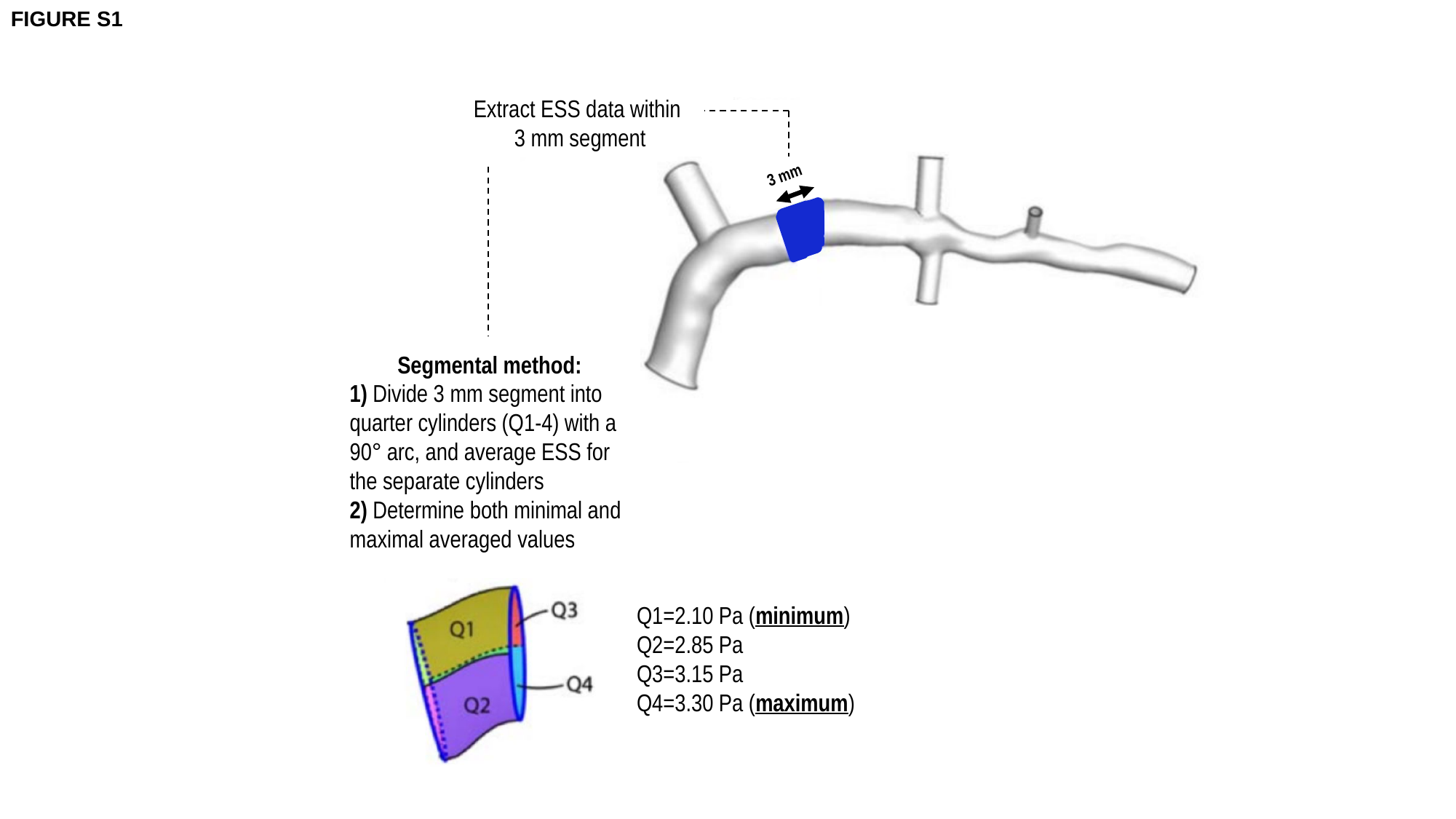

FIGURE S1
Extract ESS data within
3 mm segment
3 mm
Segmental method:
1) Divide 3 mm segment into quarter cylinders (Q1-4) with a 90° arc, and average ESS for the separate cylinders
2) Determine both minimal and maximal averaged values
Q1=2.10 Pa (minimum)
Q2=2.85 Pa
Q3=3.15 Pa
Q4=3.30 Pa (maximum)

## Slide 3
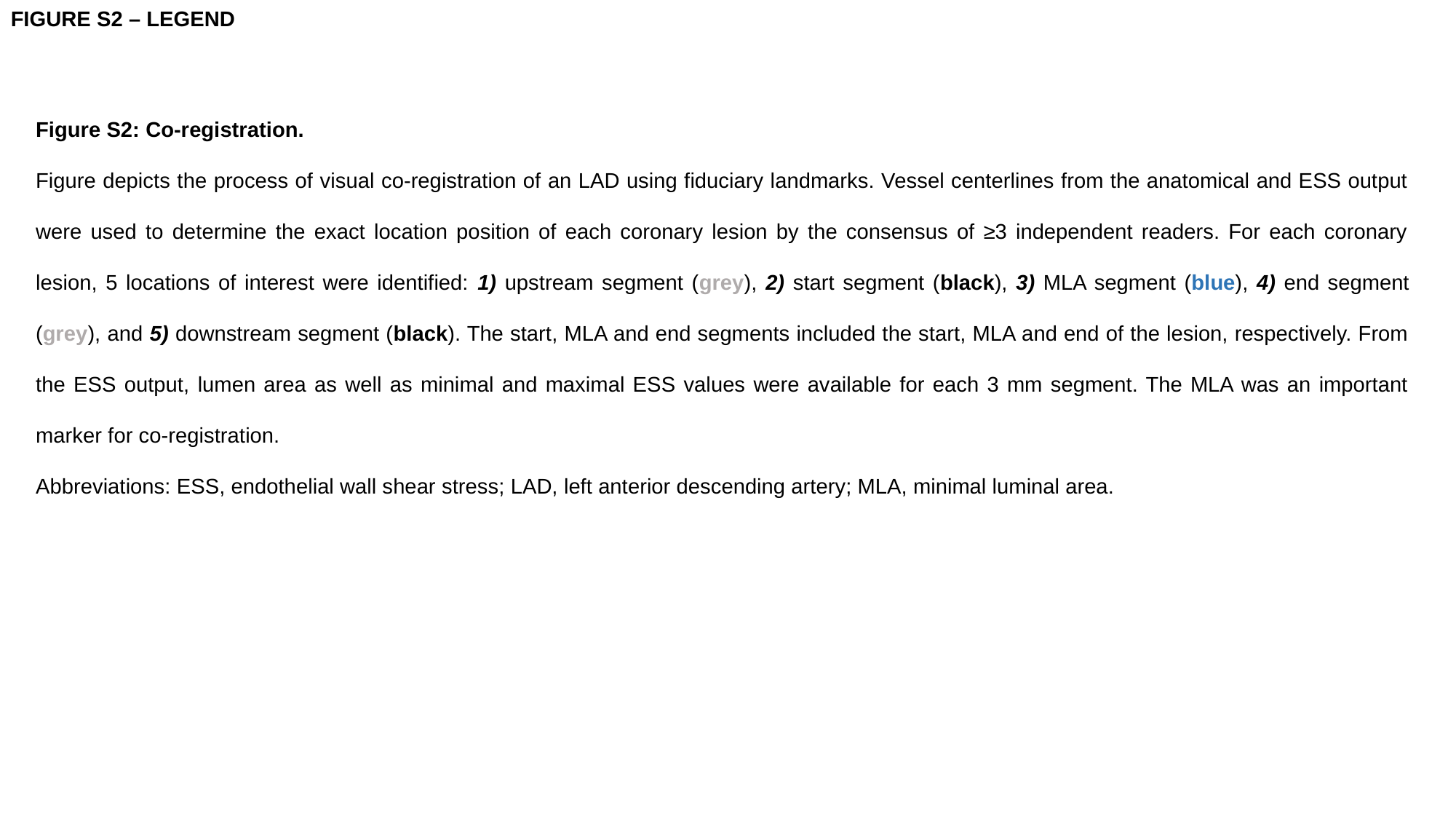

FIGURE S2 – LEGEND
Figure S2: Co-registration.
Figure depicts the process of visual co-registration of an LAD using fiduciary landmarks. Vessel centerlines from the anatomical and ESS output were used to determine the exact location position of each coronary lesion by the consensus of ≥3 independent readers. For each coronary lesion, 5 locations of interest were identified: 1) upstream segment (grey), 2) start segment (black), 3) MLA segment (blue), 4) end segment (grey), and 5) downstream segment (black). The start, MLA and end segments included the start, MLA and end of the lesion, respectively. From the ESS output, lumen area as well as minimal and maximal ESS values were available for each 3 mm segment. The MLA was an important marker for co-registration.
Abbreviations: ESS, endothelial wall shear stress; LAD, left anterior descending artery; MLA, minimal luminal area.

## Slide 4
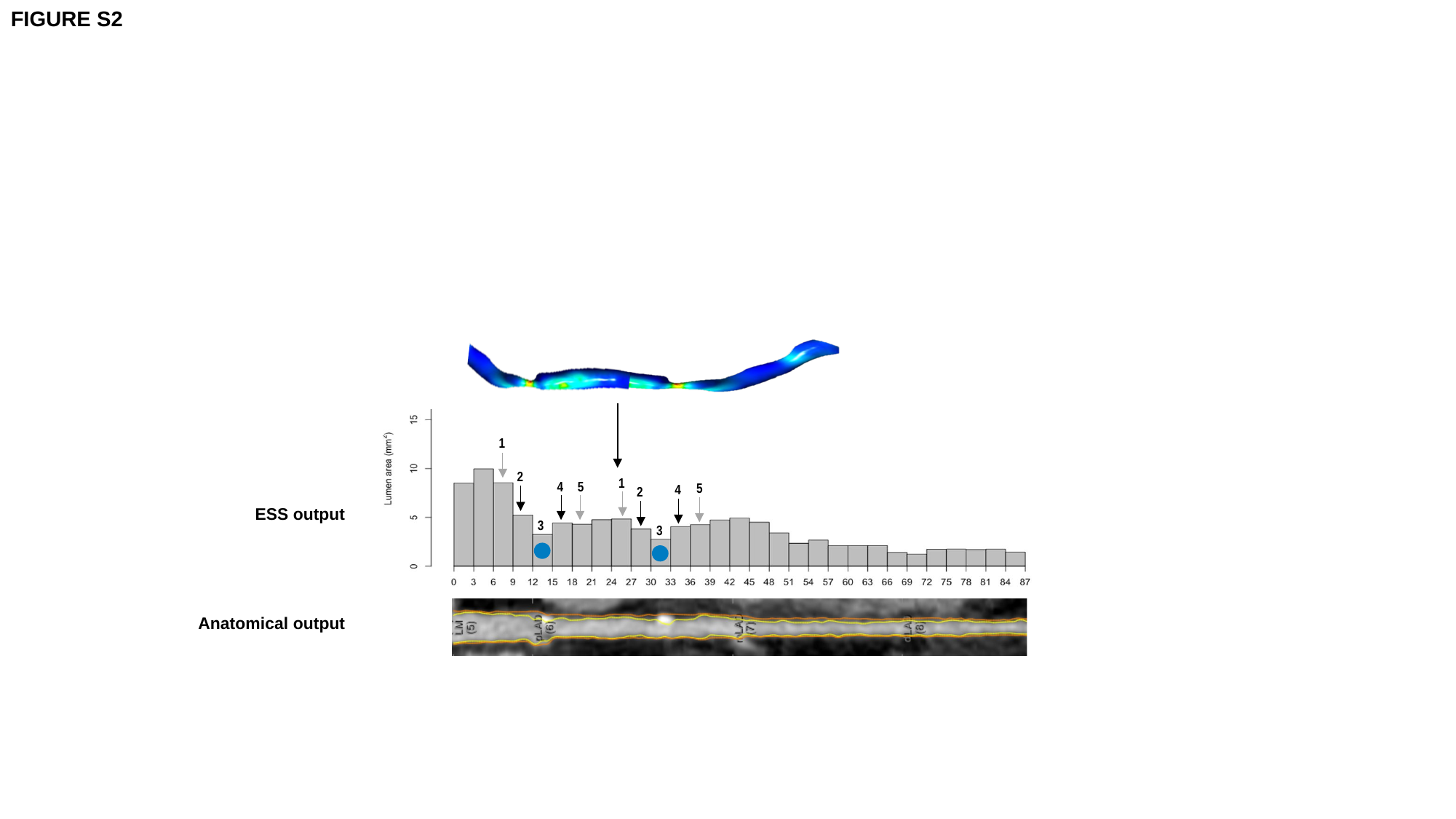

FIGURE S2
1
2
1
4
5
5
4
2
ESS output
3
3
Anatomical output

## Slide 5
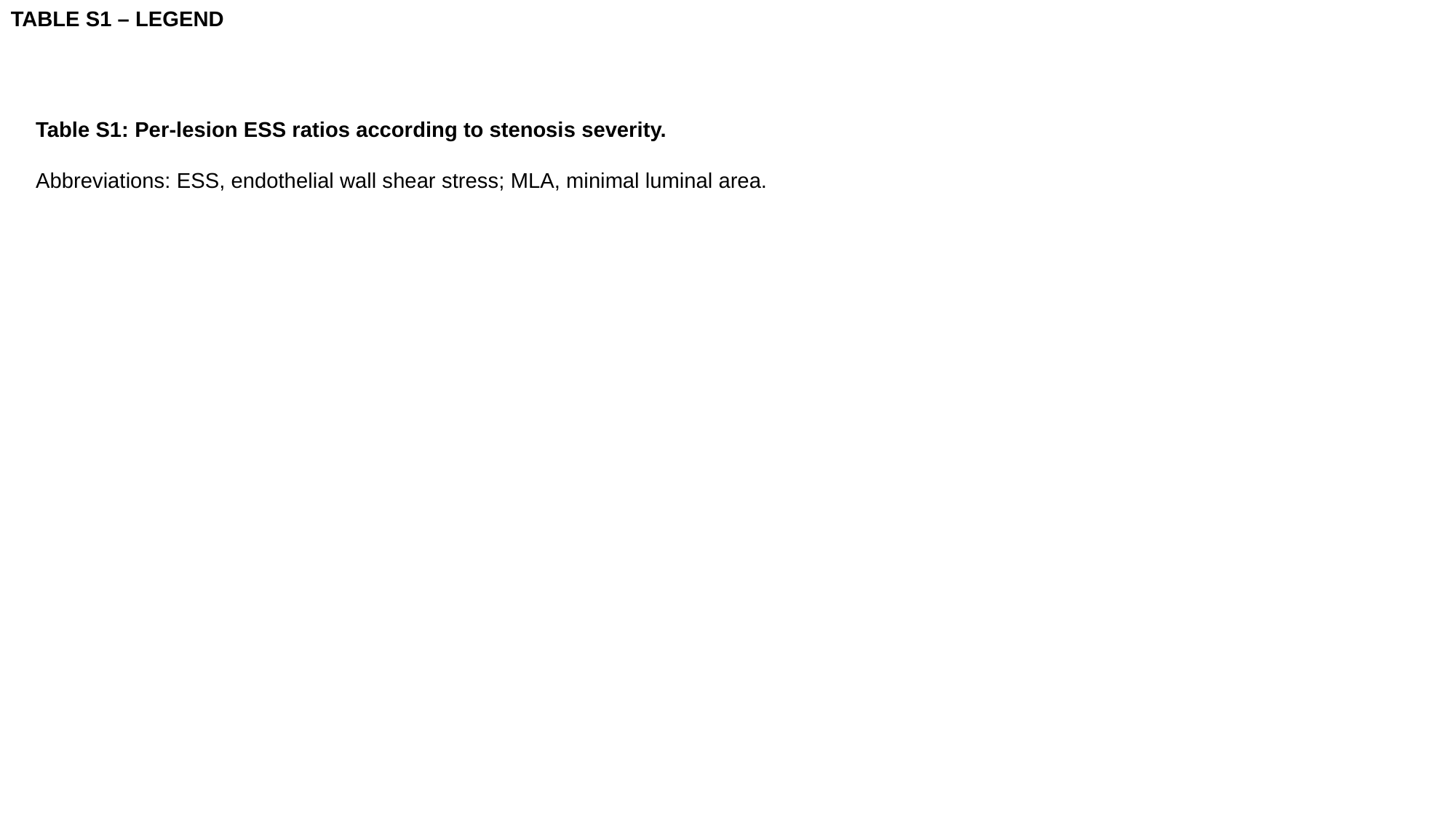

TABLE S1 – LEGEND
Table S1: Per-lesion ESS ratios according to stenosis severity.
Abbreviations: ESS, endothelial wall shear stress; MLA, minimal luminal area.

## Slide 6
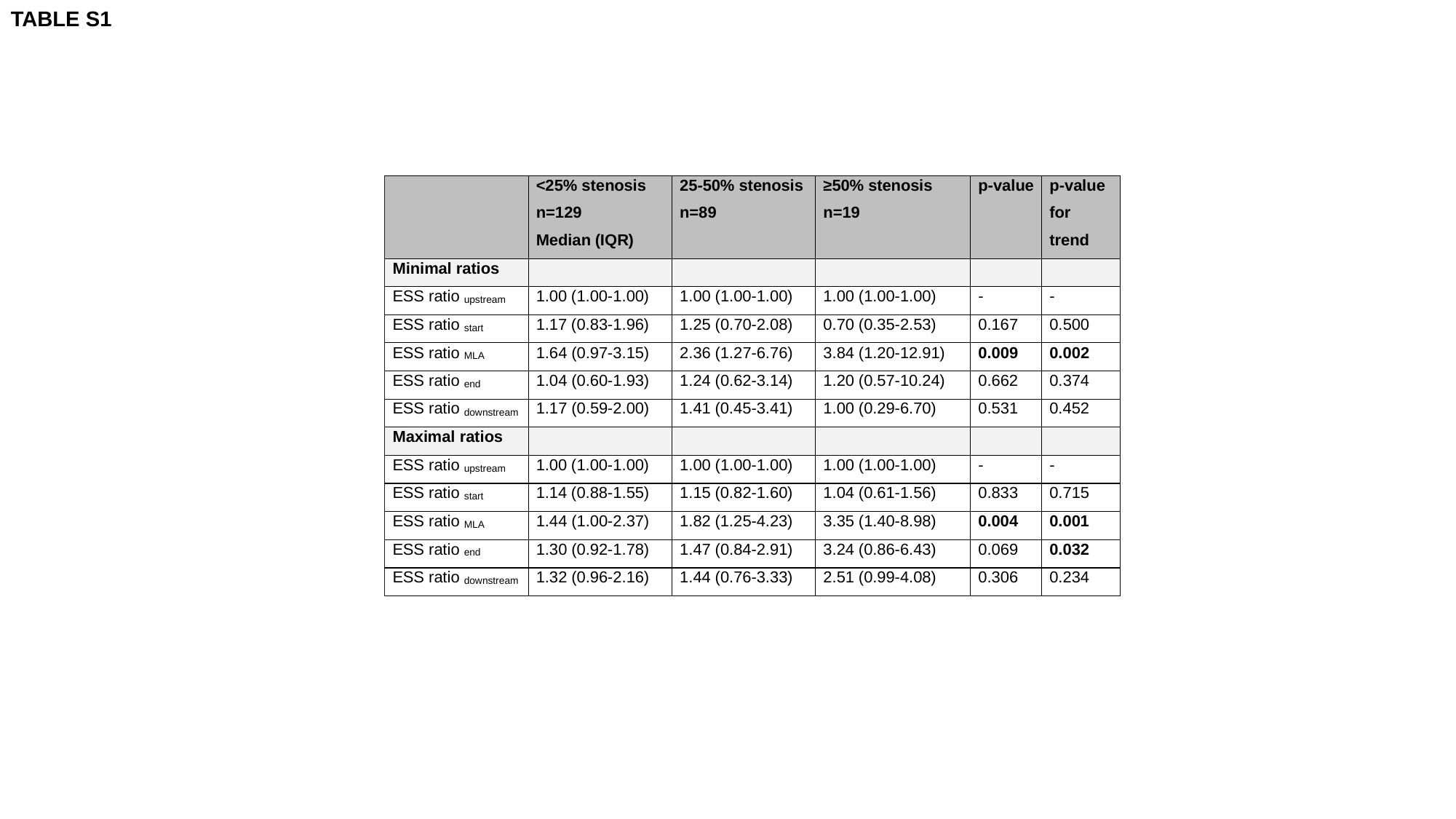

TABLE S1

## Slide 7
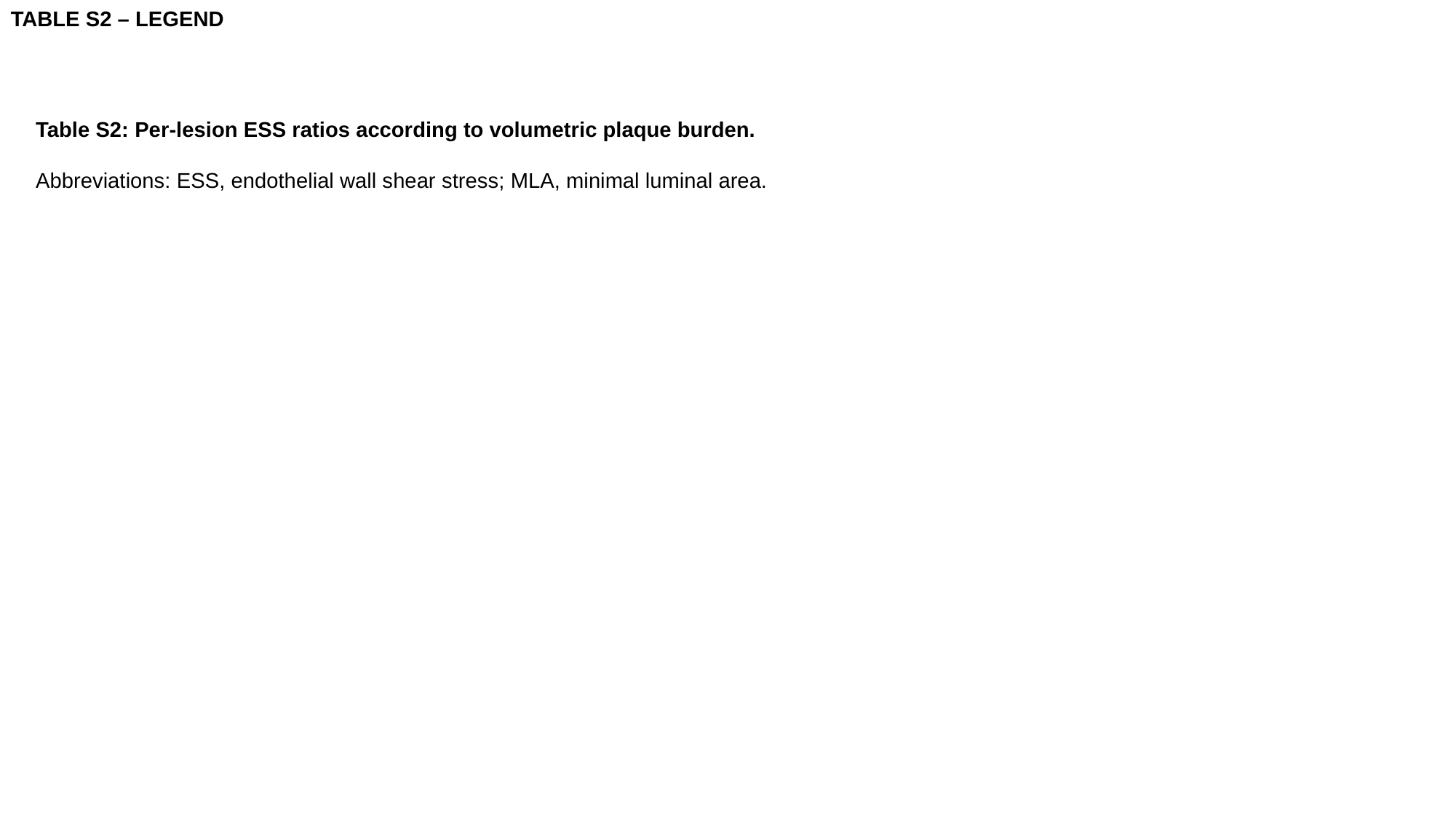

TABLE S2 – LEGEND
Table S2: Per-lesion ESS ratios according to volumetric plaque burden.
Abbreviations: ESS, endothelial wall shear stress; MLA, minimal luminal area.

## Slide 8
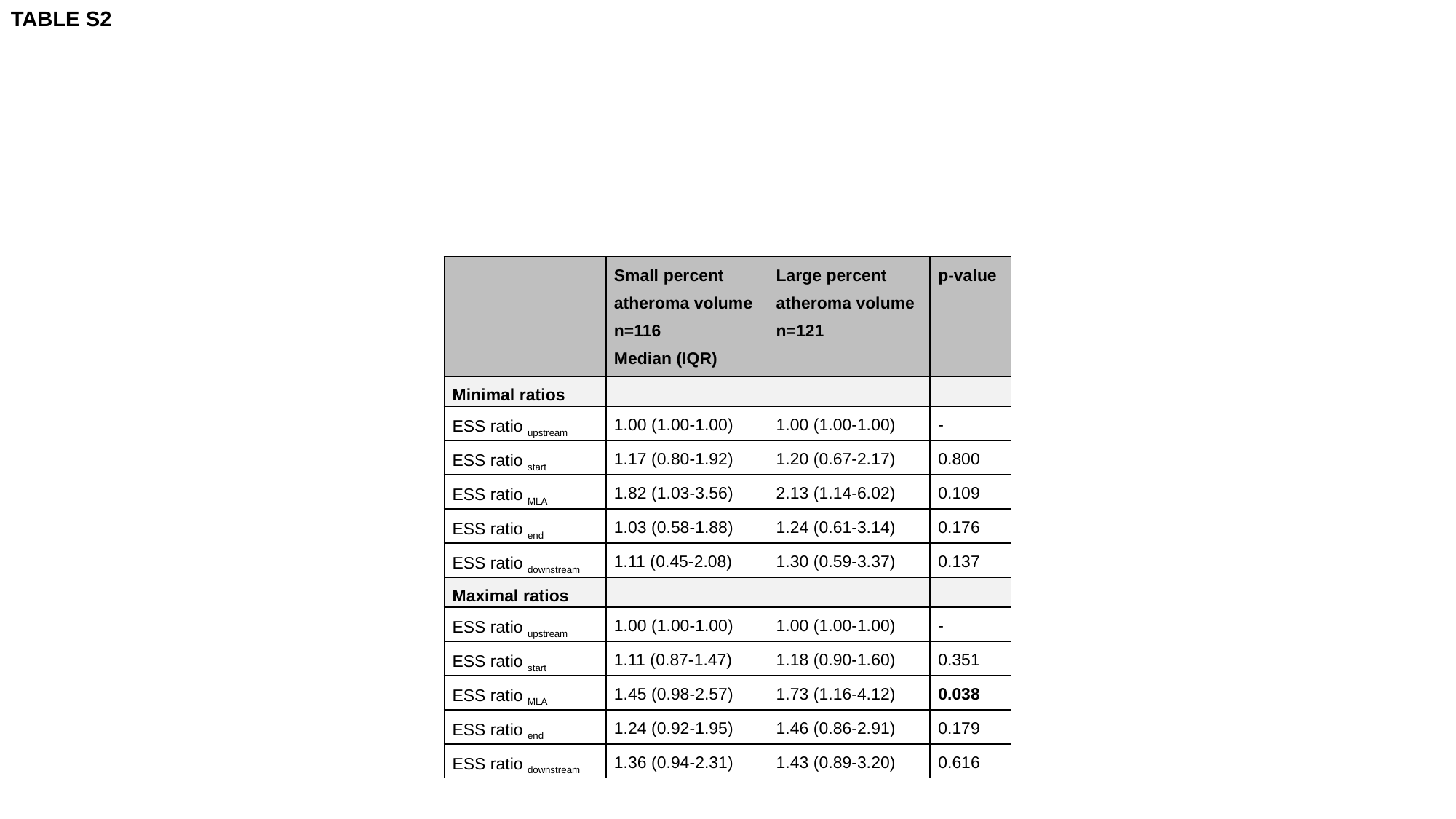

TABLE S2
| | Small percent atheroma volume n=116 Median (IQR) | Large percent atheroma volume n=121 | p-value |
| --- | --- | --- | --- |
| Minimal ratios | | | |
| ESS ratio upstream | 1.00 (1.00-1.00) | 1.00 (1.00-1.00) | - |
| ESS ratio start | 1.17 (0.80-1.92) | 1.20 (0.67-2.17) | 0.800 |
| ESS ratio MLA | 1.82 (1.03-3.56) | 2.13 (1.14-6.02) | 0.109 |
| ESS ratio end | 1.03 (0.58-1.88) | 1.24 (0.61-3.14) | 0.176 |
| ESS ratio downstream | 1.11 (0.45-2.08) | 1.30 (0.59-3.37) | 0.137 |
| Maximal ratios | | | |
| ESS ratio upstream | 1.00 (1.00-1.00) | 1.00 (1.00-1.00) | - |
| ESS ratio start | 1.11 (0.87-1.47) | 1.18 (0.90-1.60) | 0.351 |
| ESS ratio MLA | 1.45 (0.98-2.57) | 1.73 (1.16-4.12) | 0.038 |
| ESS ratio end | 1.24 (0.92-1.95) | 1.46 (0.86-2.91) | 0.179 |
| ESS ratio downstream | 1.36 (0.94-2.31) | 1.43 (0.89-3.20) | 0.616 |

## Slide 9
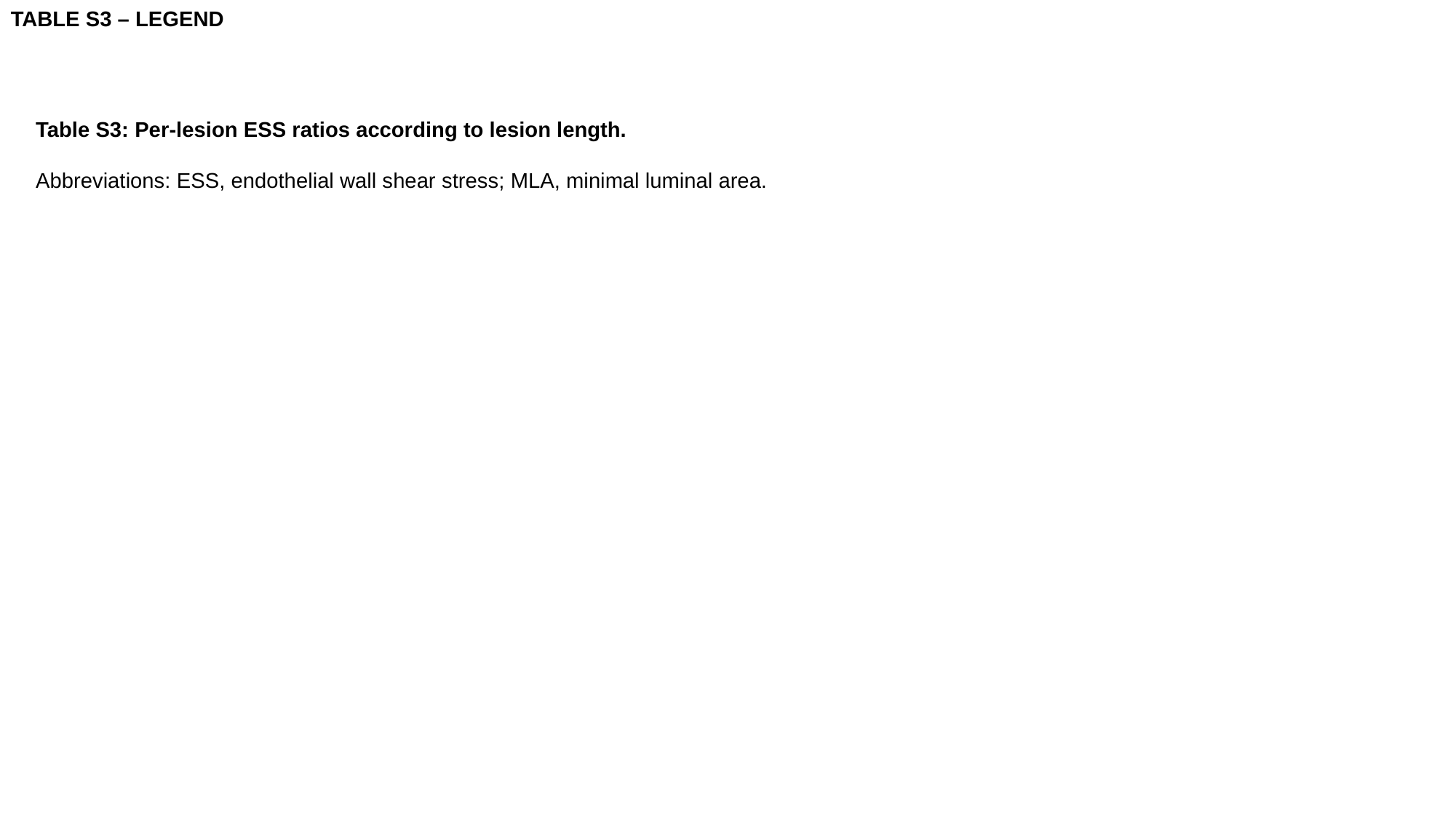

TABLE S3 – LEGEND
Table S3: Per-lesion ESS ratios according to lesion length.
Abbreviations: ESS, endothelial wall shear stress; MLA, minimal luminal area.

## Slide 10
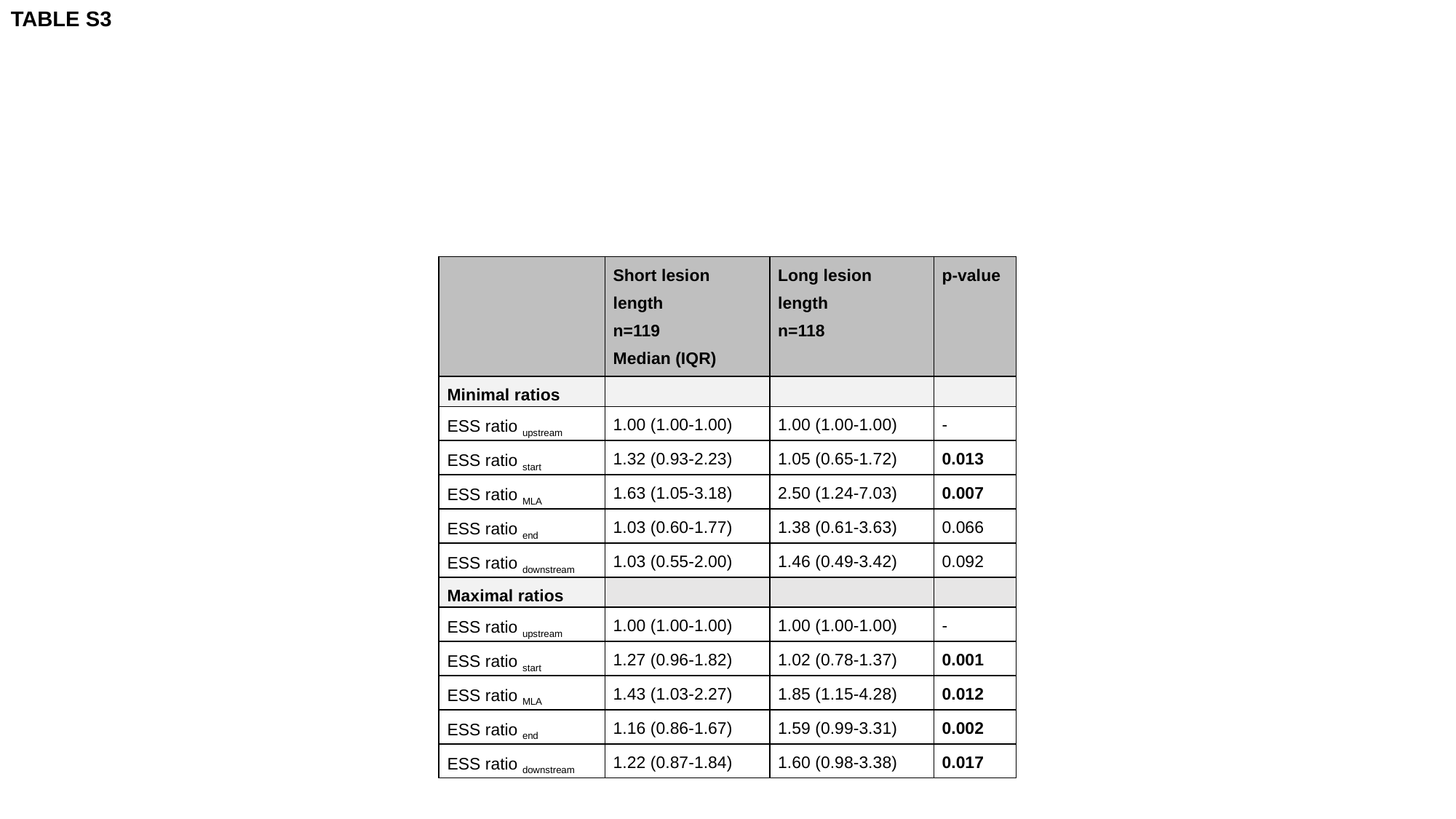

TABLE S3
| | Short lesion length n=119 Median (IQR) | Long lesion length n=118 | p-value |
| --- | --- | --- | --- |
| Minimal ratios | | | |
| ESS ratio upstream | 1.00 (1.00-1.00) | 1.00 (1.00-1.00) | - |
| ESS ratio start | 1.32 (0.93-2.23) | 1.05 (0.65-1.72) | 0.013 |
| ESS ratio MLA | 1.63 (1.05-3.18) | 2.50 (1.24-7.03) | 0.007 |
| ESS ratio end | 1.03 (0.60-1.77) | 1.38 (0.61-3.63) | 0.066 |
| ESS ratio downstream | 1.03 (0.55-2.00) | 1.46 (0.49-3.42) | 0.092 |
| Maximal ratios | | | |
| ESS ratio upstream | 1.00 (1.00-1.00) | 1.00 (1.00-1.00) | - |
| ESS ratio start | 1.27 (0.96-1.82) | 1.02 (0.78-1.37) | 0.001 |
| ESS ratio MLA | 1.43 (1.03-2.27) | 1.85 (1.15-4.28) | 0.012 |
| ESS ratio end | 1.16 (0.86-1.67) | 1.59 (0.99-3.31) | 0.002 |
| ESS ratio downstream | 1.22 (0.87-1.84) | 1.60 (0.98-3.38) | 0.017 |
